# Chemical Proprieties of Biopolymers (Chitin/Chitosan) and Their Synergic Effects with Endophytic *Bacillus* Species: Unlimited Applications in Agriculture

**DOI:** 10.3390/molecules26041117

**Published:** 2021-02-20

**Authors:** Rkhaila Amine, Chtouki Tarek, Erguig Hassane, El Haloui Noureddine, Ounine Khadija

**Affiliations:** 1 Plant, Animal and Agro-Industry Productions Laboratory, Department of Biology, Faculty of Sciences, University Campus, Ibn Tofail University, BP 133, Kenitra 14000, Morocco; Khadija.ounine@uit.ac.ma; 2 Superior School of Technology, University Campus, Ibn-Tofail University, BP 242, Kenitra 14000, Morocco; tarek.chtouki@uit.ac.ma (C.T.); hassane.erguig@uit.ac.ma (E.H.); 3 Materials and Subatomic Physics Laboratory, Department of Physics, Faculty of Sciences, University Campus, Ibn-Tofail University, BP 133, Kenitra 14000, Morocco; 4 Biology and Health Laboratory, Department of Biology, Faculty of Sciences, University Campus, Ibn Tofail University, BP 133, Kenitra 14000, Morocco; noureddine.elhaloui@uit.ac.ma

**Keywords:** chitin, chitosan, shrimp shells, *Bacillus* sp., endophytes

## Abstract

Over the past decade, reckless usage of synthetic pesticides and fertilizers in agriculture has made the environment and human health progressively vulnerable. This setting leads to the pursuit of other environmentally friendly interventions. Amongst the suggested solutions, the use of chitin and chitosan came about, whether alone or in combination with endophytic bacterial strains. In the framework of this research, we reported an assortment of studies on the physico-chemical properties and potential applications in the agricultural field of two biopolymers extracted from shrimp shells (chitin and chitosan), in addition to their uses as biofertilizers and biostimulators in combination with bacterial strains of the genus *Bacillus* sp. (having biochemical and enzymatic properties).

## 1. Introduction

Owing to their “green” characteristics, such as biodegradability, biocompatibility, renewal and durability, chitin and chitosan have progressively established notable uses in agriculture [1].

Considering that chitin is a very copious natural polysaccharide following cellulose, this polymer is attained by chemical extraction from the exoskeletons of Crustaceans, Molluscs and Arthropods [2]. Nonetheless, its derivative, chitosan is portrayed as a linear and semicrystalline polysaccharide built by glucosamine (C_6_H_13_NO_5_) and N-acetylglucosamine linked by β(1–4) glycosidic bonds, which varies from the chitin polymer by the presence of free amino groups on the second carbon atom on the D-glucose unit, rather than acetamide groups [3].

Due to the existence of the free amine group, chitosan is soluble in slightly acidic aqueous solutions, which are frequently used in agricultural applications. Chitin can also be partially depolymerized to oligosaccharide derivatives of varying chain length, or even totally depolymerized to N-acetylglucosamine [4].

Empirical assays on cultivated plants have revealed that chitin and its coproducts can be used as plant disease control agents (fungicide), fertilizers (soil conditioning agents), bactericide, nematicide, and antiperspirant. Chitin and its coproducts improve or induce natural defense mechanisms in the plant [5] and are recognized as plant growth stimulants, elicitors for the production of secondary metabolites, growth regulators and antistress agents [6].

Furthermore, numerous studies have exhibited that the use of endophytic bacteria has positive effects on stimulating plant growth through diverse mechanisms, synthesizing growth hormones (phytohormones) and augmenting the availability of nutrients to plants (biofertilizers) [7,8].

What is more, emblematic examples of biocontrol and biofertilization agents are strains of the genus Bacillus which boost the growth and development of plants via phytohormones (gibberellins, ethylene, auxins, and cytokines) and enzymes (1-Aminocyclopropane-1-carboxylic acid (ACC), chitinase and cellulase…). In addition, these strains bolster the mineralization of nutrients (solubilization of zinc, phosphate, and potassium, etc.), nitrogen fixation and a high absorption capacity of the roots [9].

Correspondingly, the combination of chitin/chitosan and endophytic strains is very useful for both; inspiring growth and immunizing plants against biotic diseases [10].

The aim of this review is to exhibit the chemical proprieties, current applications in agriculture, and future prospects for the use of chitin and chitosan with strains of the genus *Bacillus* sp. as biofertilizers.

## 2. Chitin and Chitosan

### 2.1. History of the Chitin and Chitosan’s Discovery

For the first time Chitin was isolated from a fungus in 1811 by Professor Henri Braconnot. After that, chitin’s derivative, called chitosan in 1870, was discovered in 1859 by Roughet after transformation of chitin in water soluble [11].

### 2.2. Origin of Biopolymers (Chitin/Chitosan)

Chitin is a principal component of the exoskeletons of Molluscs, Crustaceans and Arthropods. It is also found in the structure of the cytoplasmic membranes of certain fungi and algae. Chitin form approximately 14–35% of crustacean shells, as for the rest, they contain protein and calcium carbonate (Table 1) [12].

### 2.3. Characteristic and Composition of the Crustacean Shells’ Waste

Currently, crustaceans are the main accessible sources of chitin for industrial processing. The yearly production of this polysaccharide in marine ecosystems and freshwater is estimated at around 1600 and 600 million tons, respectively [14]. Amidst the prime sources of chitin are shells (including krill, crabs, lobsters, and shrimp), squid and oysters, harvested in quantities of roughly 29.9, 1.4 and 0.7 million tons per year [15]. The content of chitin in crustaceans is typically between 2% and 12% of the total body mass. This amount is gauged solely in a limited number of crustaceans. The content of chitin, proteins, carotenoids, and minerals in shell wastes fluctuate predominantly depending on the processing conditions, the part of the organism, the species, the stage of reproductive cycle and the state of their nutrition. Crustacean shells consist essentially of protein (30–40%), chitin (13–42%), and mineral salts (50%) [16]. The mineral salt contents change extensively depending on the reproductive cycle of the animals and age; hence, they influence the rigidity and permeability of the shell. Older specimens have a moderately lower percentage of chitin and a more calcified exoskeleton. The mineral fraction of the shells contains mainly phosphates, calcium and magnesium carbonates. Ergo, the presence of small proportions of lipids in the shell wastes may be the origin of viscera or the retained muscle residue. The dispersion of fatty acids in crustacean lipids is rarely reported, because of how complicated it is to study all the variations in these data generated by the impact of the ecosystems, marine, freshwater, temperature, crustacean species (and their maturity), feeding conditions, harvest season, repository and treatment history.

Apart from of the above-mentioned influences, crustacean fat always has a nutritionally valuable proportion between saturated and unsaturated fatty acids. For instance, the lipid fraction of snow crab shell waste collected in cold water includes saturated compounds (17.0–18.1%), monoenes (50.0–55.8%) and polyenes (28,2–32.0%). Additional important composites in the shell are carotenoids that are linked first and foremost with proteins in the epithelial layer of the exoskeleton. The level of carotenoids in crustaceans is especially low and diverges depending on the availability of food pigments, the size of the organism, its maturation and genetic differences. For example, the average values of the pigments’ concentrations measured in the waste (head, feet, tail) of shrimp (*P. borealis*) and crabs (*C. opilio*) were estimated at 14.7% and 13.9%, in that order [17]. The major elements of the carotenoid portion of the crustacean exoskeleton are astaxanthin and its esters, that are present in a full amount of approximately 25 μg/g of shellfish [17].

The chitin content in a dry mass of crab waste (13–26%) is lower than that of shrimp (14–42%) and krill shells (34–49%) [18]. Crab parts encompass chitin, with amounts parallel to that of Louisiana crayfish (*Procambarus clarkii*). Moreover, the mineral content of shrimp and dry crab shells is 33% and 66%, respectively.

The mineral portion of snow crab shells (*Chinoecetes opilio*) encloses 14.9% of calcium and 2.9% phosphorus. The quantity of Sr, Mn, K, and Na do not surpass 1% of the total shell mass. Though the Mn, Zn, As, Fe, Cu, and Ba elements are present in Antarctic krill (*Euphausia superba*), which serve as yet another auspicious resource of chitin [19].

### 2.4. Structure of Chitin and Chitosan

#### 2.4.1. Chitin

**Figure 1:**
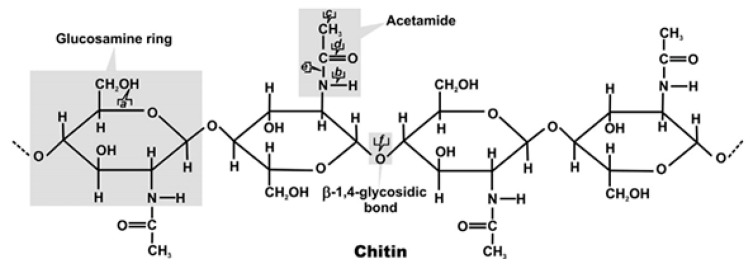
Chemical structure of chitin [20].

After cellulose, chitin is the most copious biopolymer in nature. The latter is formed by residues of N-acetyl-D-glucosamine associated by β (1→4) bonds (Figure 1).

Chitin ensures a service similar to that of plants’ cellulose, i.e., serving as a supportive and protective material. With its crystalline structure, and its connection with proteins from the exo- and endo-cuticle, it averts the internal organs of crustaceans from swelling in seawater [21].

It is well established that chitin has a very systematized and arranged crystal structure that can be in different polymorphic forms (α, β and γ), which differ from each other according to the chain arrangement in the crystal region. The α chitin is the most profuse form and evidently the most stable one, since β and γ chitins can be converted into α chitin by appropriate treatments [22].

**Figure 2:**
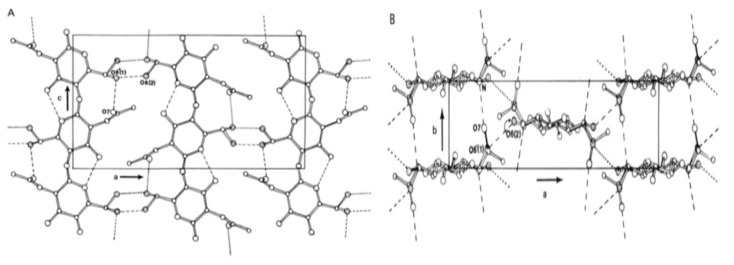
Structure of α chitin. (**A**) ac projection; (**B**) ab projection. The branching of the atoms indicates the alternate positions for the oxygen of the CH_2_OH group [23].

The α-chitin is by far the most prevalent stature present in Arthropods, Crustaceans, fungi, and yeasts. It is the most stable structure thanks to the hydrogen and covalent bonds between the chains (Figure 2). The glycosidic bonds are aligned in a di-axial geometry, thus implying a diagonal position of the units of N-acetylglucosamines. This bolsters the lateral association of hydrogen bonds between chains and C=O groups to N–H groups [3].

**Figure 3:**
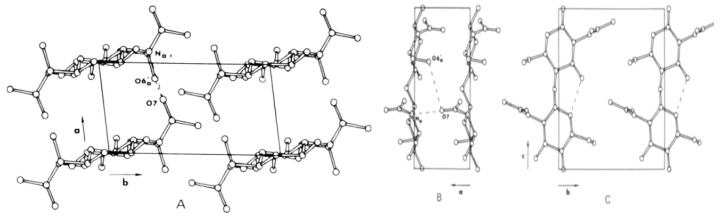
Structure of β chitin. (**A**) ab projection; (**B**) ac projection; (**C**) bc projection [24].

Contrariwise, the β chitin is less steady; less crystalline and scarce (Figure 3). It is present in squid plumes, in tubes produced by worms of the pogonophores and vestimentifera family, in certain algae and protozoa [3].

#### 2.4.2. Chitosan:

**Figure 4:**
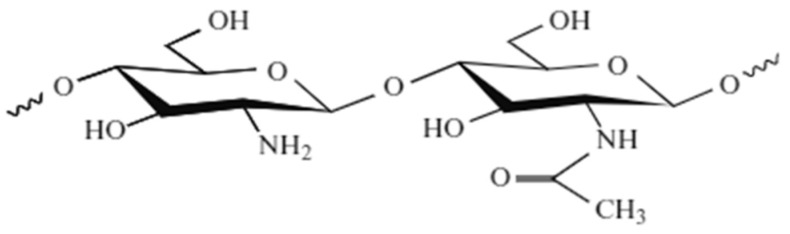
Chemical structure of chitosan [26].

Chitosan is an N-glucosamine units polymer linked in ß-(1–4) (deacetylated unit) and N-acetyl-D-glucosamine (acetylated unit) [25] (Figure 4).

Chitosan is originated by enzymatic or chemical deacetylation of chitin, the substance of which ranges from 44% for the arthropods exoskeleton (crustaceans), 40% for the cephalopods endoskeleton (squid) and 42% for the chassis of the fungi. The conversion of chitin to chitosan requires primordial steps, which start with demineralization (with HCl), deproteinization (NaOH or KOH) and ultimately decolorization [27].

Chitosan has a level of acetylation that fluctuates from 5% to 30%, with a molecular weight (MW) between 1 and 12 × 10^4^ Da (from fungal mycelia), and about 1.5 × 10^6^ Da (from crustaceans) [28]. It can manifest in an amorphous, a semicrystalline or a crystalline form. Chitosan can form bonds with negatively charged molecules, such as fatty acids, phospholipids, proteins, bile acids, polysaccharides like pectins and alginates [29].

#### 2.4.3. Chemical Properties of Chitosan

Molecular mass: molecular mass (MM) has an important part in the solubilization and viscosity of chitosan solutions. It can be measured by the size-exclusion chromatography (SEC) coupled with detectors such as refractometer and multiangle light scattering. This method allows determining the average molecular mass in both molecular number (Mn) and molecular mass (Mw) in addition to the mass distribution in the sample [30].

The chitosan’s molecular mass value depends scrupulously on the manufacturing process, seeing as the latter can induce depolymerization of the macromolecular chains and/or degradation during its production. The chitosan chains have incredibly elevated masses (up to 1–3 Mda in case of products extracted by supervised processes of extraction and deacetylation but ordinarily they are lower, averaging between 100 and 1500 kDa). As matter of fact, these masses depend on the type of the desired application: e.g., for applications in the field of water treatment, the molecular mass (MM) of chitosan is generally amid 100 and 500 kDa. Indeed, it is not required to use chitosan with high molecular mass (MM) to have interesting results [31].

The molecular mass affects the solubility of chitosan and its rheological properties, predominantly its viscosity. For example, the high viscosity of the solution could bind the possibility of handling concentrated solutions as well as obtaining materials with high chitosan density. It is then a matter of accomplishing an accommodation between the rigidity of the system (favored by high mass chitosan) and the rheological properties of the solution (easier handling for low mass polymers). The demonstration of behavior is linked to the critical concentration of the crisscrossing that is inversely proportional to the mass of the polymer. This is the limit concentration at which the polymer tends, by folding, to form additional inter- and intrachain hydrogen-type bonds [31].

Deacetylation degree: by definition, deacetylation is the removal of an acetyl group (COCH_3_) from a molecule (Reaction 1), this degree of deacetylation (DD) can be used to set apart chitosan and chitin, as it determines the content of amine groups free in biopolymers. This bestows considerable weight to the degree of deacetylation, because it affects the physicochemical properties of chitosan [32]. Deacetylation also affects immunological activity and biodegradability [13].

R–NHCOCH_3_ + OH^−^ → R–NH_2_ + CH_3_COO^−^ (1)

Chitosan has two advantages over chitin. The first is connected to dissolution, because chitin is dissolved in highly toxic solvents such as dimethylacetamide and lithium chloride, whilst, chitosan is effortlessly dissolved in acetic acid. The second advantage is that chitosan has free amino groups that represent active sites for many chemical reactions [33].

Depending on its degree of deacetylation, chitosan is classified as having a low degree of deacetylation (DD) in the deacetylation range from 55% to 70%, medium, 70–85%, high, 85–95%, and ultra-high between 95% and 100% [34,35]. A variety of methods have been characterized in order to determine the degree of deacetylation (DD) of chitosan. These include ninhydrin, linear potentiometric titration, near infrared spectroscopy, infrared spectroscopy, hydrogen bromide titrimetry, nuclear magnetic resonance spectroscopy and ultraviolet–visible derivative spectrophotometry (UV) [36]. The infrared spectroscopy (IR) method, first proposed by Moore and Roberts (1978) [37], is frequently used for estimating the degree of deacetylation (DD) values of chitosan. This technique has a number of pros and cons. First and foremost, it is somewhat fast and unlike other spectroscopic methods, does not necessitate the purity of the sample to be tested nor the dissolution of the chitosan sample in an aqueous solvent [38]. Nevertheless, the IR method uses the baseline for the calculation of DD; on the other hand, there may perhaps be a possible quarrel for using different baselines which would inevitably contribute to differentiating DD values. Secondly, the sample preparation, the type of instrument used, and the conditions can greatly affect the analysis of the sample. Seeing that chitosan is hygroscopic in nature, samples with lower DD can soak up more moisture than those with higher DD, it is vital that the analyzed samples are completely dry [36].

**Biodegradation:** chitosan is vulnerable in front of the hydrolytic activity of various enzyme systems such as chitinase, chitosanase, dextranase, pectinase, lipase, pepsin, papain, pancreatin, and lysozyme [39], whereas only chitinase, chitosanase and lysozyme are considered specific. Chitosan is more susceptible to the nonspecific action of certain enzymes than it was supposed. Chitosan can be deteriorated by α–amylases in plants, fungi and humans. It is also sensitive to the action of lipases in animals and plants [39].

The effect of chitosanase on the glycosidic bonds of chitosan, prompt the production of glucosamines and heterooligosaccharides, which consist of glucosamines and N-acetyl-D-glucosamine [40].

**Figure 5:**
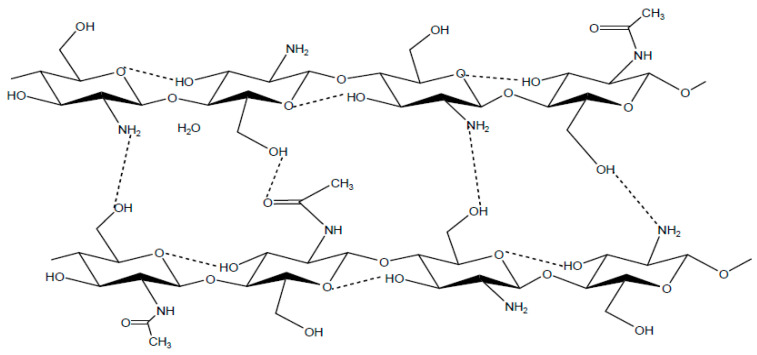
Crystal structure of chitosan [43].

**Solubility:** below its pKa (pH = 6.5), chitosan is easily soluble in dilute acidic medium. This solubility is mainly due to the ability of the amino groups in the chitosan backbone to increase ionization by forming chit-NH^3+^ at low pH, therefore the solubility increases [41] (Figure 5).

**Figure 6:**
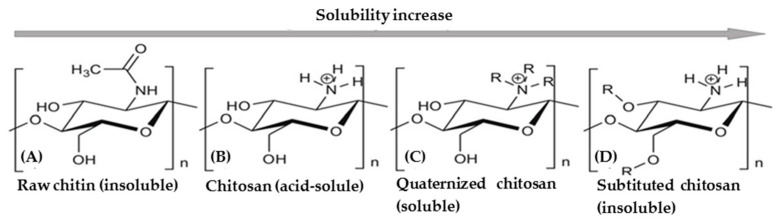
Depiction of chitin and chitosan by functionalization of the chemical structure and the solubility of the biopolymer. (**A**) Raw chitin (insoluble), (**B**) acid soluble chitosan, (**C**) quaternized chitosan (soluble), and (**D**) substituted chitosan show much better solubility under alkaline conditions than chitosan [43].

Another important property of chitosan is its degree of acetylation (DA), which affects other properties such as solubility, crystallinity and hydrophobicity [42] (Figure 6).

### 2.5. Global Demand and Production of Chitin and Chitosan

It is increasingly obvious that the universal demand for chitin and chitosan is becoming substantial, in view of the fact that in 2015, it reached more than 60,000 T, yet the world production of that same year did not exceed 28,000 tons. While, the predictions delivered in the Global Industry Analysts report (chitin and chitosan derivatives market report—2015), foresees a surge reaching up to USD 63 billion in the global market of these derivatives.

At this stage, the production of chitin and chitosan relies on crab shells and shrimp disposed by preservation industries. Various countries have remarkable untapped crustacean resources, e.g., Chile, Norway and Mexico [44]. The production of chitosan from crustacean shells acquired as waste from the food industry is economically feasible, especially if it incorporates the retrieving of carotenoids. The shells hold substantial quantities of carotenoid, astaxanthin that are not synthesized, and which are marketed as a nourishing additive for fish in aquaculture, particularly for salmon [45].

For the sake of producing 1 kg of 70% deacetylated chitosan from shrimp shells, 6.3 kg of HCl and 1.8 kg of NaOH are needed, coupled with nitrogen, process water (0.5 t), and cooling water (0.9 t). The most essential items in estimating the cost of production include transportation, which differ in accordance with the job and location. In India, the Central Institute of Fisheries Technology (CIFT) of Kerala has launched a number of studies on chitin and chitosan. Based on their inquiry, they discovered that the dried shrimp waste held 23% chitin as for the dry squilla it contained 15% [46]. The international price for chitosan (in small quantities) is USD 7.5/10 g (Sigma-Aldrich price list).

### 2.6. Methods of Gauging Crustacean Coproducts:

#### 2.6.1. Chemical Method

The production of chitin and chitosan is based on the purification of the raw material. The general idea of this treatment relies on eradicating the calcium carbonate and proteins to have the chitin and the acetyl group.

In the early-stage, prepping the raw material is critical to cut down the hazards of deterioration of chitin. The methods are specific to each producer, frequently based on drying to avoid natural autolysis [11]. As an exemplification, the manufacturer France-chitine uses salting to preserve shrimp shells (*Parapenaeopsis stylifera*) for a long time, specifically during their transport [47].

Chemical extraction consists of an acid treatment for demineralization and an alkaline treatment for deproteinization and deacetylation. The steps of this protocol, which will be detailed underneath, have been the topic of several optimizations implemented by researchers specializing in the field. More often than not, the isolation of chitin is achieved by means of demineralization, deproteinization, and bleaching. The first two steps can be used in reverse order, depending on the method of carotenoid, protein recuperation and the chitin application [48].

**Pretreatment:** the target of this particular step is to get rid of all impurities from the shells before the grinding and processing. For this purpose, two types of courses have been suggested; one involves washing with tap water [49], as for the other, it advocates boiling the shrimp shells in water for 1 h to remove excess tissue and then place them in an oven at 163 °C for 1 h [50].

**Demineralization:** the demineralization of shrimp shells demands the addition of an acidic solution, in particular hydrochloric acid.

As reported by Truong et al. (2007) [27], concentrations of 2 M HCl were added to the powder of the shrimp shells, with a ratio of 1:10 *w/v* (weight/volume), then, the mixture was left for 3 h at a temperature of 50 °C. On the other hand, the team of Charoenvuttitham et al. (2006) [49] used a concentration of approximately 0.25 M HCl for 30 min and at room temperature, to finally attain an extraction percentage of 28.8 ± 1.7%.

**Deproteinization:** by default, the waste from the shrimp shells is deproteinized by aqueous NaOH or KOH solutions. The effectiveness of alkaline deproteinization is contingent on the treatment temperature, the solution/shell ratio and the concentration of the base. Crustacean wastes are frequently treated with dilute sodium hydroxide solution at concentrations ranging from 1% to 10% (*w/v* “weight/volume”) and at elevated temperature (65–100 °C) [48].

**Decolorization:** carotenoids withdrawal from chitin can be acquired by extraction at room temperature with ethanol, acetone, ethyl acetate, chloroform and/or ether mixture. The decolorization is regularly conducted by a bleach treatment with NaOCl and H_2_O_2_ solutions [48].

**Deacetylation:** this step’s principle is based on the hydrolyzation of N-acetyl linkages with aqueous solution of sodium hydroxide (NaOH). This chemical reaction occurs at high temperatures, with continuous agitation and during periods varying according to the protocols innovated by the researchers.

Kurita et al. (2003) [51] discovered that, upon deacetylation of chitin from shrimp shells, prolonged treatment causes extensive degradation of major chitin chains without a significant increase in deacetylation.

#### 2.6.2. Biological Method

Several studies have exposed the importance of fermentation in processing crustacean coproducts. In fact, this method is based on the production of exocellular proteases via certain microorganisms, along with the production of acidic ionic species (such as lactic acid) that promotes the extraction of chitin.

The fermentation stages are held in a reactor where the temperature, pH, pressure, and agitation conditions are closely monitored. Subsequent to the fermentation, a filtration separates two fractions. The solid fraction is composed chiefly of chitin, whereas the liquid fraction encompasses the other solubilized components. One of the perks of the biological process is the potential value of this last fraction [11].

### 2.7. Application of Chitin

#### 2.7.1. Applications in the Agricultural Sector

For quite some time, chitin and chitosan have been deemed to be evokers and elicitors in plants. They entail the promoting of the production of secondary metabolites that fortifies the plants immune defense mechanisms. Chitin derivatives, for example, stimulate the production of phenylalanine ammonia-lyase (PAL) and tyrosine ammonia-lyase (TAL) that are considered to be the two central enzymes in the phenylpropanoid pathway, which itself intervenes in the responses to the biotic and abiotic factors [52]. Another analysis executed by Dörnenburg and Knorr in 1994 [53], once more attests that chitosan and chitin induce the biosynthesis of anthraquinone (a compound of the family of polycyclic aromatic hydrocarbons) that plays an important role in the protection procedure of plants.

#### 2.7.2. Chitin’s Effect on Crop Protection

Chitin and its coproducts have been used to shield crops against disease before or after harvest, directly or indirectly, depending on the specific interaction of the plant pathogen [4].

#### 2.7.3. Antifungal Activity of Chitin

Chitin ensures the protection of plants through two core mechanisms:

- Direct action on the molecules of the fungus affecting their growth and development.
- Triggering the defense mechanisms that interfere with or hold back the development of pathogens, which consequently halt or limit the progression of the disease.

The use of chitin as a nutrient is very common in bacteria. Namely: *Pseudomonas*, *Vibrionaceae, Photobacterium, Enterobacteriaceae, Actinomycetes, Bacillus*, and *Clostridium* [54]. On top of that, this polymer exhibits behaviors on fungal species, such as slime molds, Chytridiomycetes, Zygomycetes, Deuteromycetes, Ascomycetes, and Basidiomycetes [54].

### 2.8. Applications of Chitosan

Countless industries exploit chitosan, like agriculture, paper, textiles, water treatment, pharmacy, medical devices. The food industry capitalizes on its antibacterial and antifungal properties to reduce the use of synthetic preservatives. Nowadays, its use as a nutritious additive is best known as “fat blocker” given that chitosan inhibits the metabolization of fats thanks to the interactions between its amine functions and the carboxylic groups of lipids [55]. Its superior chelating properties allow applications in the reprocessing of wastewater while its free amino groups are in fact capable of binding all transition metals and radionuclides [56].

The exploitation of chitosan in the agriculture field displays effects on acceleration of plant growth and improvement of crop yields. Ultimately, its many biological properties make it a suitable candidate of choice for biomedical applications: antimicrobial agent, hemostatic, healing dressing, etc. It is also vigorously studied as a restricted release system of therapeutic agents by oral, transdermal, ocular, and nasal routes [57]. It certainly has good mucoadhesive properties and an absence of toxicity offering, thereby, uses in gene therapy and in vaccination. Conversely, this polymer does not display any antigenic behavior, but has an antithrombogenic and hemostatic character, along with remarkable healing properties [58]. Chitosan has in addition the ability to hinder the growth of many parasites and bacteria. It also has immunological, antitumor, antibacterial, and antifungal properties [59]. Furthermore, it causes the activation of macrophages and stimulates the production of circulating antibodies.

The efforts of Bacon et al. (2000) [58] indicates that the utilization of chitosan in the case of the influenza B/Panama virus, gives a strong immunogenicity stimulation compared to the natural reaction [58]. Trials by Devlieghere et al. (2004) [60] explain that chitosan acts on the bacterial wall by weakening it until it breaks. Chitosan therefore appears to have a lytic action on bacteria and certain encased viral agents [60].

#### 2.8.1. Antiviral Activity

Given that viruses exploit cellular mechanisms for their own benefit in order to reproduce, the strategies to fight against these distressing enemies of plants remain delicate and deterrent.

Following the same pathway as the chemical pesticides currently in use, chitosan has demonstrated a strong protection of plants against contamination agents, e.g., potatoes inoculated with virus X illustrate a strong resistance to this virus following spraying with 1 mg/mL of the chitosan solution having different molecular weights (3,36 and 120 kD) [61].

The potential explanation for this mechanism is largely rooted in the fact that chitosan induces a hastening of plants. Chirkov et al. (2001) [61] noted a boost in the callose content and the level of ribonuclease activity in plants treated with chitosan, before their infections by the PVX virus (Potato Virus X).

#### 2.8.2. Nematicidal Activity

On the same species, *Meloidogyne incognita* (causative agent of root gall in tomatoes), two different teams of researchers Khalil and Badawy (2012) [62] and Mohamed et al. (2012) [63], tested the beneficial effects of chitosan on this problematic species under greenhouse conditions and in vitro. These studies revealed that the molecular weight 2.27 × 10^5^ g/mol, enabled a better inhibition of the second larval stage of *M. incognitain* in vitro. As a consequence, under greenhouse conditions, the application of chitosan (molecular weight 2.27 × 10^5^ g/mol and 3.60 × 10^5^ g/mol) shows a significant reduction in egg mass and the number of tomato root galls in soil infested with *M. incognita* [62]. Additionally, the study carried out by Mohamed and his collaborators (2012) [63], noted that an amendment of the soil by chitosan reduces the number of galls of the tomato roots with 72.03%, hence, it inhibits 69.87% of the larvae at the 2nd juvenile stage.

#### 2.8.3. Antioxidant Activity

Thanks to the use of the free radical DPPH (2,2-diphenyl-1-picrylhydrazyl) at 0.02%, Rajalakshmi et al. (2013) [64] were able to identify the antioxidant activity of chitosan at a concentration of 1 mg/mL of solution.

Eventually, these authors [64] stated that the extracted chitosan reveals antioxidant and free radical trapping activity, coupled with activity towards DPPH, hydrogen peroxide, and superoxide anion radicals. Regardless, they cited as perspectives that the antioxidant activity in vivo and the different antioxidant mechanisms need to be studied in greater depth.

#### 2.8.4. Antifungal Activity

Chitosan is also renowned for its antifungal properties in opposition to an assortment of fungi with the exception of those enclosing chitosan as an important component of their cell casing, such as zygomycetes [31].

Chitosan has been revealed to be useful in combating phytopathogenic strains such as *Colletotrichum gloeosporioides* [65], *Aspergillus niger* [66], *Rhizoctonia solani*, *Fusarium solani* and *Sclerotium rolfsii* [67], *Fusarium oxysporum* f.sp. radicis-lycopersici, *Penicillium digitatum* [68], *Pyricularia grisea* [69].

#### 2.8.5. Antimicrobial Activity

The antimicrobial effects of chitosan have been recorded in several early and current studies. Contingent on the concentration, the molecular mass, the degree of deacetylation, the type of bacteria, the pH, and the conditions of application, chitosan demonstrates different activities. The following paragraph recaps some studies that have been done with the purpose of proving this activity.

On four species of Gram-negative bacteria (*Pseudomonas fluorescens, Escherichia coli, Vibrio parahaemolyticus*, and *Salmonella typhimurium*) and seven Gram-positive bacteria (*Staphylococcus aureus, B. cereus, Bacillus megaterium, Listeria monocytogenes, Lactobacillus plantarum, L. bulgaricus*, and *L. brevis*). No et al. (2002) [70] have exposed by the use of six types of chitosan of different molecular weights that the antimicrobial effect is observed past 0.1% of chitosan towards Gram-positive bacteria compared to Gram-negative bacteria and that this activity is conversely impacted by pH (pH 4.5–5.9).

In accordance with the same subject, Tsai et al. (1999) [71] tested the effect of the age of the bacterial culture, the pH, the temperature, the concentration of chitosan (98% deacetylated), and the complex associations of the latter with other ions on the bactericidal activity of chitosan against *E. coli* in vitro. The obtained results lay bare that the age of the bacterial culture influences the bacteria’s sensitivity to chitosan (the late exponential phase cells are more sensitive to chitosan). A higher temperature (25 and 37 °C) and an acidic pH augment the bactericidal effects of chitosan. Sodium ions (Na^+^) at 100 mM could complicate chitosan and consequently reduce its activity (Na^+^ and chitosan form a complex which reduces binding to the cell surface). Divalent cations at concentrations of 10 and 25 mM diminish the antibacterial activity of chitosan, of the order of Ba^2+^—Ca^2+^—Mg^2+^.

Limam et al. (2010) [72] elucidated this mechanism of bacterial growth inhibition, by the fact that the cationic charge of the amine group, can combine with anionic components such as, neuraminic acid, sialic acid and N-acetylmuramic acid from the cell surface, and can hinder bacterial growth by impaired exchange with the medium, chelation of nutrient transition ions and inhibition of enzymes.

#### 2.8.6. Plant Growth Stimulating Activity

The implemented endeavor by the various researchers confirms the advantageous effect of chitosan on growth and flowering is constantly applied to monocotyledons and dicotyledons. As a case in point, Khin and his collaborators (2006) [73], assert that the chitosan enables growth acceleration 15 times more than the control group of the meristematic tissues of orchid cultivated on liquid medium and on solid medium. Similarly, the obtained results by Boonlertnirun et al. (2008) [74] testify that the application of chitosan by amending the soil and soaking the seeds (four times throughout the growing season), enhanced the rice yield dramatically, compared to other treatments (application of chitosan by foliar spraying and soaking the seeds). Proportionately, the collected results on the tomato corroborate this effect, since the spray treatment of chitosan allows an enhance in all the agronomic parameters of the plant, specifically, the weight, the number of leaves and the number of branches, compared to the organic fumigation and treatment with synthetic amino acids [75].

Chitosan’s application touches a broad array of parameters like morphology (height of the plant, number of the plant leaves), growth (absolute growth rate, total growth rate, relative mass of the plant), yield (number and size of fruits), and biochemical parameters (nitrate reductase and photosynthesis) [76]. On the scale of secondary metabolites of plants, Kim et al. (2005) [77] who worked on basil (*Ocimum basilicum* L.) insist that the amount of phenolic and terpene compounds grew after chitosan treatment, quintessentially; the amounts of rosmarinic acid (RA) and eugenol (4-allyl-2-methoxyphenol). Not only that, the growth in terms of weight and height of the basil drastically increased by approximately 17% and 12%, respectively, compared to the control.

Complementary results are gathered by Liopa-Tsakalidi et al. (2010) [78] on medicinal plants; namely *Melissa officinalis*, *Artemisia dracunculus.* These researchers concluded that the use of chitin (2 g/L) affects the length, fresh or dry weight of the stem as well as the root of these two plants. Moreover, in terms of plant biochemistry, chitin did stimulate the percentage of chlorophyll (a) with 47% and by 60% for chlorophyll (b) in these two plants. This noteworthy increase in the concentration of chlorophyll shows a great ability of chitosan to improve the performance of photosynthesis which generates a better yield of plants [79].

The upgrade in the “in vivo” and “in vitro” agronomic parameters is explained by the fact that chitosan and its derivatives are composed of nitrogen; the latter only becomes absorbable by plants through microbial decomposition that releases inorganic nitrogen or via direct assimilation of monomers as organic nitrogen. Other than that, the application of chitosan increases the absorption of mineral nutrients such as phosphorus, potassium, nitrogen, magnesium, and calcium, which stimulates the growth of plants, treated with chitosan [79]. Other researchers have established that the chitosan application also increased the number of stomata as well as stomatal conductance. In view of its polycationic nature, it stimulated the level of ABA, resulting in higher conductance and accumulation of CO_2_ in the cell, resulting in a pronounced effect on yield [80,81].

The functional groups of chitosan (hydroxyl and amino groups) allow the formation of complexes with ions of iron, zinc, copper, and others. This makes chitosan a sustainable alternative to synthetic chelating agents. The cationic property of chitosan also makes it a proper carrier for the deliverance of additional essential nutrients. Chitosan augments the anion exchange capacity of the soil (AEC) and as a result lessens the leaching of nutrient anionic fertilizers such as nitrates and phosphates from soils. Aside from the regulated release of nutrients, chitosan polymers have also been used effectively to improve the delivery of certain pesticides in crops in order to better their efficiency and reduce their impact on the environment [82].

Seeds soaked in chitosan unveiled an upturn in the germination rate, length, and weight of hypocotyls and radicle in rapeseed. It is also deemed to be beneficial in improving the germination rate of cabbage, pumpkin, chili, and cucumber. Coating the seeds with chitosan boosts germination and potency of *Pennisetum glaucum* seedlings. Aside from that, it has been pointed out that treating seeds with acidic solutions of chitosan ameliorates the vigor of corn [83].

In our recent study, the obtained results provide evidence that chitosan and chitin–chitosan mixture have an important role in the promotion of *Lycopersicon esculentum* L., *Capsicum annuum* L. and *Solanum melongena* L. seed germination percentage by 16%, 34% and 22%, compared to the control. Thus, treating the seeds with 25, 50 and 100 mg/L of chitin or chitosan results in an improvement of the vigor index, shoot and root lengths of these three species. The in vivo tests showed that weekly soil amendment with chitin or chitosan induced the stimulation of plants parameters (lengths, fresh and dry weights of aerial and root part). However, a very significant increase in the number and weight of fruits is marked by the weekly soil amendment with the chitin–chitosan mixture at 25 and 100 mg/L [84].

#### 2.8.7. Stimulating Activity of the Plant’s Defense

In the interest of preventively sensitizing plants against attacks by phytopathogens, techniques of protection that are based on the utilization of the natural defenses of plants by the use of an elicitor (a molecule of diverse origin triggering the defense mechanisms of plants with the production of defensive substances) have been cultivated.

The labor of an ample assortment of scientists has shed light on the resistance-inducing characteristics of chitin and chitosan. This opened the way for the possibility of using these nonphytotoxic compounds [85] as a biological control agent in agriculture and horticulture [86].

Furthermore, the earlier study by Rkhaila and Ounine, (2018) [87] validates the effectiveness of the application of 100 mg/L chitosan in being the most successful in inhibiting the mycelial growth of *Fusarium oxysporum* f.sp. radicis-lycopersici (FORL) in vitro, whereas in vivo they have made evident that the weekly amendment of tomato plants (*Lycopersicon esculentum* L.) with chitin at 25 mg/L minimizes the symptoms due to FORL attacks, wherefore the plants treated with this biopolymer record the minimum value of the leaf stunting index.

#### 2.8.8. Complex Associations of Metal Ions: Essential Role in Water Purification

Appertaining to the chelating property of chitosan, Rhazi, (2002) [88] and his collaborators performed an experiment that consists of mixing metal ions with a chitosan solution, which made it possible to categorize the metal ions according to their affinities with the chitosan:

Cu^2+^ ≥ Hg^2+^ > Zn^2+^ > Cd^2+^ > Ni^2+^ > Co^2+^, Ca^2+^ (2)

Eu^3+^ > Nd^3+^ > Cr^3+^ > Pr^3+^ (3)

These sequences are not contingent on the physical shape of the chitosan. In addition, this selectivity seems to be independent of the size and hardness of the considered ions [88].

## 3. Endophytic Bacteria

The term “endophyte” originates from the Greek words “endon” signifying “internal”, and “phyton” meaning “plant”. Earlier, endophytes were defined as microorganisms like bacteria and fungi that inhabit the radices of plants for all or part of their life cycle without causing noticeable damage to the host plant [89].

The micro-organisms isolated from the internal plant tissue of healthy plants incorporate more than 129 species representing more than 54 genera, *Bacillus*, *Enterobacter*, *Pseudomonas*, and *Agrobacterium* being the frequently isolated bacterial genera [90].

Not long ago, Hardoim et al. (2015) [91] defined endophytes as microbes including archaea, fungi, protists, and bacteria that colonize the interior of the plant, in spite of the association result.

In reliance on lifestyle, endophytic bacteria can be distinguished as [92]:

- Obligatory endophytes bank entirely on the host plant for their survival and growth. Their transmission to other hosts is carried out either vertically or by vectors.
- Facultative endophytes have a stage in their life cycle where they exist outside of the host plants. At the extremity, phytopathogenic bacteria could be included as endophytes (facultative or obligatory).

For instance, *Ralstonia solanacearum biovar* 2, which can survive in water systems, can behave as endophytic bacteria, in an apparently avirulent form, inside tomato plants. Avirulent forms of phytopathogens should consequently be considered as endophytes, while virulent forms should not be included [92].

**Figure 7:**
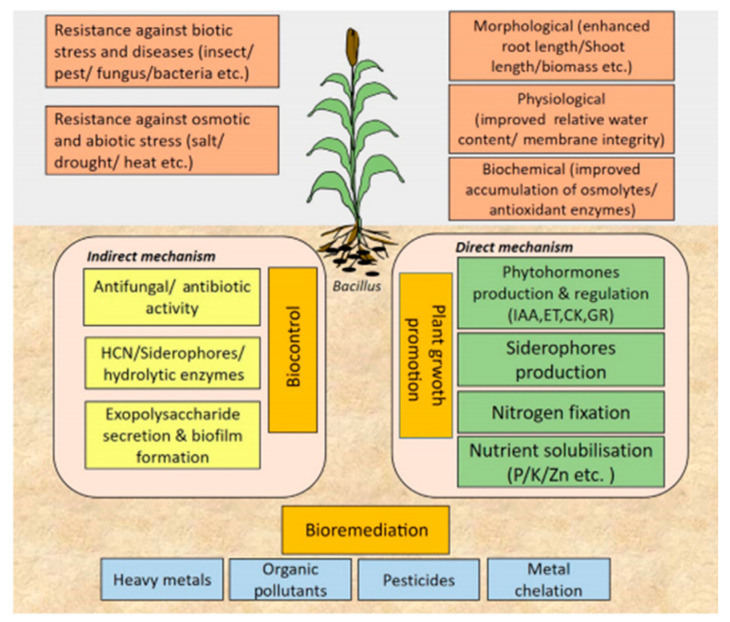
Root colonization process of endophytic bacteria of the genus *Bacillus* sp. [97].

Inside the plants there is a microbial diversity explained by the ability of various endophytes to penetrate and persist in plants [93]. These endophytes more often than not originate from the soil, primarily infecting the host plant by colonizing, as a case in point, cracks created in lateral root junctions, and then quickly spreading to the intercellular spaces of the root [94]. Even though there are alternative doorways of entry into the plant, for example, injuries caused by microbial plant pathogens or nematodes or stomata found in leaf tissue [95]. On the flip side, root cracks are recognized as the main “hot spots” of bacterial colonization [96] (Figure 7).

### 3.1. Endophytic Bacteria of the Genus Bacillus sp.

The *Bacillus* genus refers to the family *Bacillaceae*. It stands out from other family representatives by loads of characters. They are bacilli with square or rounded ends, of variable size (0.5–1.2 µm and 2.5–10 µm “diameter and length”), Gram-positive, dexterous of producing endospores (a form of resistance and a taxonomically significant criterion of the genus *Bacillus*), they are bacteria that are cultivated aerobically (aeroanaerobic, or strictly aerobic), mobile due to a peritrichous ciliature [98].

The *Bacillus* genus is profoundly heterogeneous and encircles a hefty number of species. This genus appears to be tremendously heterogeneous on the phenotypic genetic level (respiratory type, sugar metabolism, composition of the wall, habitat, etc.). Their taxonomy is far from being simple, because under this name a substantial number of very different Gram-positive bacilli are gathered together [97].

This convoluted situation shows the adversities encountered in identifying sporulating bacteria, the interest of which is above all industrial. The most used classification is based on the shape of the spore, differentiating in the process three groups:

- Bacilli with nondeforming oval spore;
- Bacilli with oval deforming spore;
- Bacilli with round deforming spore.

Many studies have determined the advantageous effects of endophytes, by showcasing their benign effects in radically improving the yield and growth of numerous types of crops [99].

In reference to bacteria affiliated with the *Bacillus* genus, Xie et al. (1998) [100] uncovered the activity of the nitrogenase enzyme in B. *subtilis*, B. *cereus*, B. *megaterium*, B. *circulans*, B. *licheniformis*, B. *pumilus*, B. *firmus*, and B. *brevis.* These authors recorded in 2003 the isolation of 14 strains of *Bacillus* capable of reducing acetylene in rice fields at eight sites on the banks of the Yangtze River in China.

Li et al. (1992) [101] had also identified a species of *Bacillus* which fixed nitrogen in connection with ecto-mycorrhizae, meanwhile Ahmad et al. (2008) [102] identified isolates of *Bacillus* that fastened nitrogen in diverse rhizosphere soils in Aligarh-India. *Bacillus fusiformis* (strains PM-5 and PM-24) was also classed as a nitrogen fixing bacteria using the acetylene reduction test. This species has been shown to display intense nitrogenase activity in different plant crops in Chungbuk Province, South Korea. *Bacillus diazotrophs* have even been found in the rhizosphere of pines and oaks [103]. Ding et al. (2005) [104] identified a biological activity of nitrogen in B. *marisflavi* and *Paenibacillus massiliensis* and also identified fragments of the nifH gene in *Bacillus megaterium, Bacillus cereus*, and *Bacillus alkalidiazotrophicus* [105]. Therefore, the two genera, *Bacillus* and *Paenibacillus*, have been shown to have species capable of fixing nitrogen. Fascinatingly, *Natronobacillus*, a new genus of bacillus, was created specifically for N. *azotifigens*, which is an anaerobic *Bacillus alkalidiazotrophicus* bacteria isolated from salt-rich habitats [106]. These types of bacteria play an important role in keeping soil fertile. A huge hurdle for the development of sustainable agriculture lies in the use of nitrogen fixing bacteria capable of assimilating gaseous N_2_ from the atmosphere [107].

Beneduzi et al. (2008b) [108] researched a number of bacilli strains, fundamentally species of the genera *Bacillus* and *Paenibacillus*, presenting important characteristics (PGPR “Plant Growth-Promoting Rhizobacteria”) isolated in seven unmistakable rice production areas of the Rio Grande do Sul state, south of Brazil. From these 296 isolates, 94 and 148 produced between 0.1 and 30 mg.mL^−1^ of indole-3-acetic acid (IAA) in vitro after 72 and 144 h of incubation, in that order. Twenty-two isolates were able to solubilize the phosphate and 32 isolates produced siderophores. The genera *Paenibacillus* and *Bacillus* were the ultimate important groups in the rhizosphere and soil populations analyzed. *Paenibacillus borealis* was the most frequent species in both locations. The *Paenibacillus borealis* isolate SVPR30, pinpointed by sequence analysis of the 16S rRNA gene as a strain of *Bacillus* sp., was chosen for in vivo greenhouse experiments and found to be very valuable in abetting a significant increase in roots and aerial parts of the rice plants.

In wheat crops, the genus *Paenibacillus* was the most critical group both in the rhizosphere (77.8%) and in the soil (79%). *Paenibacillus borealis* was the most frequently identified species, followed by *Paenibacillus graminis*. The remainder of the isolated bacteria belonged to the genus *Bacillus* sp. The production of the indole compounds (indole-3-acetic acid (IAA) and indole-pyruvic acid (IpyA)) was detected in 33.6% and 26% of isolates from the rhizosphere and soil, respectively. Of the 311 isolates, nine were competent at solubilizing phosphate and 48 were capable of fabricating siderophores. The SBR5, CSR16 and EsR7 isolates, identified by the 16S rRNA gene sequence as strains of *Paenibacillus* sp., were selected for in vivo greenhouse experiments and established to be very effective in stimulating a major raise in growth and dry matter of wheat plants [109].

The *Bacillus* and *Paenibacillus* strains are Gram-positive endophytes, which can be inoculated separately or in combination with *Rhizobium* or *Bradyrhizobium* strains that are Gram-negative PGPRs, to support plant growth. The inoculation of *Rhizobium* and the strain of *Paenibacillu spolymyxa* H5 (a phosphate solubilizing bacteria), brought forth higher chickpea yields due to increased absorption of phosphorus and nitrogen [110]. Colonization and nodulation of soybean with *B. japonicum* strains [111] may amplify in an environment containing *Bacillus* spp., ensuing in elevated plant dry weight and seed yields.

### 3.2. Endophytic Bacteria and Growth Stimulation

Endophytic bacteria could accelerate plant growth by directly generating phytohormones and other growth regulators such as lipochito-oligosaccharides and lumichromas along with improving host anabolism (photosynthesis) and the phytohormone levels of the plant [112].

Popular characteristics of endophytes include the aptitude to produce siderophores (enhances bacteria’s competitiveness), to synthesize plant hormones (indole-3-acetic acid), to solubilize phosphate (inorganic form), and bestow plant tolerance to abiotic and biotic stresses [113].

It has been unveiled that the endophytic bacteria *Burkholderia phytofrmans Psjn* can vitalize the cold tolerance of vine plants by changing photosynthetic activity and the metabolism of carbohydrates entangled in tolerance to cold stress [114]. The existence of bacteria in the plant helped adaptation to cooling temperatures, resulting, thus, in lower cell damage, higher photosynthetic activity, and a build-up of metabolites, such as starch, phenolic compounds and proline. Synonymous positive effects of the bacteria on metabolic balance and reducing the effect of drought stress have been evidenced in wheat plants grown under water stress conditions [115]. Endophytic bacteria *Pseudomonas pseudoalcaligenes* exposed an induction of the accumulation of glycine betaines at high concentrations in rice plants, which allows them to tolerate salt stress [116].

Over and above that, Cohen et al. (2009) [117] prove that accumulation of hormones (abscisic acid, gibberellin and IAA) produced by *Azospirillum* spp. allows tolerance to water stress in corn plants.

Abscisic acid is one of the most important phytohormones for the development and growth of plants, and its concentrations are known to increase dramatically in states of stress. The fundamental function of this phytohormone appears to be the adjustment of plant water balance and tolerance to osmotic stress [118].

What is more, the inoculation of plants with the *Achromobacter xylosoxidans* strain AUM54 displayed a surge in growth in soils containing a concentration of 150 mM of NaCl. The study by Qin et al. (2014) [118] laid bare high tolerance to salinity in halophytic plant *Limoniumsinense* inoculated with endophytic bacteria producing ACC deaminase. Bacteria with ACC deaminase can be found in approximately 13 isolates belonging to various genera: *Bacillus*, *Klebsiella, Pseudomonas*, *Arthrobacter, Serratia, Microbacterium, Streptomyces,* and *Isoptericola* [119].

#### 3.2.1. Production of Indole-3-Acetic Acid (IAA) and Other Hormones

The production of IAA is a common characteristic among endophytic bacteria, and is produced by a vast range of phylum/bacterial classes isolated from several plants, including poplar, epiphytic, cactus, soybean, orchids, strawberry, and potato. The production of IAA by endophytic bacteria is associated with the stimulation of plant root growth, increased biomass and volume of this part [120].

#### 3.2.2. Improvement of Photosynthetic Activity

Bacterial endophytes can actively alter the physiology of the host plant [93]. The introduction of three endophytic bacteria, decidedly *Chryseobacterium indologene, Acinetobacter johnsonii*, and *Bacillus pumilus* into *Beta vulgaris* upped the chlorophyll content of plants, leading to heightened synthesis of carbohydrates compared to control plants [121]. The authors perceived that the unidentified compounds produced by endophytes could have a positive effect on chloroplast metabolism by the enhancement of electron transport.

#### 3.2.3. Regulation of Ethylene Levels by the Bacteria Producing ACC Deaminase

Ethylene is a multifunctional plant hormone typically involved in fruit ripening, seed germination, the formation of root hairs and mature xylem vessels, leaf senescence and flowering. Its effects depend on the type of plant tissue, its growth state and its physiological environment. In plants, ethylene is synthesized from methionine through a dual route. This reaction is unrolled in presence of a precursor, nonproteic amino acid ACC. Several biotic and abiotic factors can induce the synthesis of ethylene and ACC. In regard to ethylene synthesis, this reaction is activated by auxins, in particular IAA and CKs, and can be inhibited by abscisic acid (ABA) [122].

A category of endophytes is able to break down the precursor (bacterial encoded) ACC deaminase and use the final products as nitrogen and carbon sources. Simultaneously; these bacteria reduce ethylene concentrations in colonized plant tissue and revive plant growth under stressful conditions [123]. Effectiveness on plant growth parameters like root elongation and escalated biomass have been ascertained by many endophytic species such as *Burkholderia* and isolates of *Pseudomonas*, *B. cepacia*, *Arthrobacter, Bacillus* spp., and *Methylobacterium fujisawaense* [124].

## 4. Chitin and Chitosan Degradation Enzymes

### 4.1. Chitinases

Chitinases play important physiological roles depending on their origin, e.g., they are capable of hydrolyzing insoluble chitin into oligo and monomers present in a range of organisms, such as bacteria, insects, fungi, viruses, higher animals, and plants ([125]; [126]). They are composed of the most popular genera, specifically *Serratia*, *Aeromonas*, *Streptomyces*, *Bacillus,* and *Vibrio* [127].

Some related polymers can be hydrolyzed by chitinases, like cell wall polysaccharides encompassing not only N-acetylglucosamine linked to β-1,4 but also N-acetylmuramate [128]. In bacteria, these chitinases are between about 20 and 60 kDa, considered analogous in size to plant chitinases (about 25–40 kDa), and comparable to insect chitinases (about 40–85 kDa) [128].

Overall, chitinases are split into three categories [129]:

- Exochitinases, that only show activity for the nonreducing end of the chitin chain;
- Endochitinases, hydrolyzing the internal β-1, 4-glycoside;
- β-N-acetylglucosaminidase, that cleaves GlcNAc units sequentially from the nonreducing end of the substrate.

Being an arbitrary splits process, the hydrolyses take place arbitrarily at internal locations along the length of the biopolymer, thusly leading to the liberation of soluble low molecular weight monomers such as, chitobiose, chitotetraose and chitotriose. These oligosaccharides then turn into a substrate for β-N acetylglucosaminidase. For bacteria, the degradation of chitin causes the transformation of the biopolymer (GlcNAc)_n_ into fructose-6-phosphate, acetate and NH_3_ [130].

Chitinase possesses a broad selection of applications, e.g., the preparation of pharmaceutically important N-acetyl D-glucosamine and chito-oligosaccharides, the isolation of protoplasts from fungi and yeasts, the synthesis of unicellular proteins, the treatment of chitinous waste, and the control of malaria transmission [131].

### 4.2. Families of Chitinases

Chitinolytic enzymes are grouped into families; 18, 19 and 20, hinged on the resemblance of amino acid sequence [132]. In terms of evolution, family 18 is differentiated and includes chitinases from certain plants, fungi, bacteria, viruses, and animals. Certain chitinases from *Streptomyces* and plant chitinases (classes I, II, and IV) are regrouped in family 19 [133]. The chitinases of the two families, i.e., 18 and 19, are likely to have evolved from different ancestors because they are composed of different amino acid sequences, 3D structures and completely different molecular mechanisms [134]. Family 20 includes β-N-acetylhexosaminidases from humans, bacteria, and Streptomyces [131].

The roles of its chitinases diverge according to the species. The digestive tract of vertebrates is the place where chitinases are produced [135]. This enzyme is dedicated to the partial degradation of old shells in crustaceans and insects; through a complex hormonal mechanism this synthesis is controlled [136]. In plants, chitinases are activated upon attack by pathogens to inhibit proteinases, glucanases and chitinases [137]. Chitinases are also present in fungi and have an autolytic, nutritional and morphogenetic role [138].

The recent classification of chitinases is founded on the mode of action, and classifies chitinases into [139,140]:

- Chitinases (EC 3.2.1.14) that cleave the chitin chain at internal sites haphazardly. They are found in four families of glycoside hydrolases (GH) (18, 19, 23, and 48).
- β-N-acetylhexosaminidases (EC 3.2.1.52) that catalyze the respective deletion of GlcNAc (N-Acetylglucosamine) residues from the nonreducing end of the chain, and they are included in GH3, GH18, GH20, and GH84.

**Bacterial chitinases:** microbial chitinases fulfill a crucial role in combating phytopathogens in plants. Class A chitinase (ChiA) is especially researched for its multiple applications in biological control. Current studies have shown an outstanding reduction in contagion of beans and cotton plants by soil fungi *Sclerotium rolfsii* and *Rhizoctonia solani* on the presence of the overproducing *Escherichia coli* strain of ChiA [141]. In addition, transgenic *Arabidopsis thaliana* containing the Chitinase 2 gene from Chinese Wild Strawberry improves resistance to anthracnose disease [142].

Bacterial chitinases are encountered in families GH18, GH19 and GH23 [143]. Yet, GH18 family includes the most bacterial chitinases [144], which are classified into three subfamilies (A, B and C), based upon sequence homology [145]. It is worth bearing in mind that this classification is not respected by the nomenclature of bacterial chitinases; since, *Bacillus circulans* chitinase (D) is classified in subfamily (B) while *Serratia marcescens* chitinase (B) belongs to subfamily (A) [146]. Certain bacterial GH18 chitinases retain fibronectin-like type III domain that plays a role in binding to the substrate, in addition to the CBM (carbohydrate-binding module) and catalytic domains [147].

Contrastingly, it has been suggested that GH19 chitinase genes have been transferred to nematodes and arthropods by purple bacteria and *Actinobacteria* who have procured it from plants [142]. Recently, only one GH23 chitinase is found in bacteria, especially it has been isolated from *Ralstonia* sp. A-471. This enzyme has homology with the lysozyme, because it includes a chitin-binding-domain (N-terminal join to a C-terminal of the catalytic domain). The proposed hypothesis suggests that the horizontal transfer of genes coding for this enzyme allowed the transfer to the other generations [148].

For some species, chitinases can play a crucial role in supplying bacteria with essential precursors or nutrients. In the spirochete *Borrelia burgdorferi*, for example, the degrading capacity of the peritrophic membranes allows it to grow in environments with low levels of unbound NAcGlc (N-Acétylglucosamine), such as in the intestinal part [149]. *Pseudoalteromonas* chitinase system has also been exhibited to be embroiled in nitrogen metabolism [150]. Nevertheless, chitinases are probably not fundamental for many other heterotrophic bacteria, as in most habitats a variety of nutritional sources are available. Furthermore, in other microorganisms called autotrophs (e.g., *Cyanobacterium Anabaena*), inhibition of the growth of competing organisms such as fungi requires the production of chitinases that are part of their allelopathic system [151].

Chitinases are also involved in bacterial pathogenesis. In cases where the host is known to contain chitin, such as in insects, the mechanism seems simple. For example, the entomopathogenic bacterium *Yersinia entomophaga* produces an ABC-type protein toxin complex that can eradicate the host within three days of infection [152]. This complex has been shown to include two subunits with chitinase activity that anchor the complex and facilitate its penetration through the peritrophic membrane [152]. Bacterial pathogens transmitted by vectors of nonchitinous organisms such as plants and mammals also produce chitinases to colonize the insect vector by digesting the peritrophic membrane [153].

**Chitosanase:** as an enzyme hydrolyzing the glycosidic links in chitosan, chitosanase (EC. 3.2.1.132) has obtained exceptional attention, due to its importance in terms of maintaining the ecological equilibrium, for recycling huge wastes of chitinous nature generated from marine species, for the enzymatic preparation of chito-oligosaccharides and for the biological control of fungal pathogens [154].

On the flip side, the use of lingo-cellulosic substrates depends on the production of oxidizing and hydrolytic enzymes capable of converting lingo-cellulosic compounds into low molecular weight molecules [155]. Cellulolytic enzymes, such as cellobiase (EC. 3.2.1.21) exo-cellobiohydrolase (EC. 3.2.1.91), and endoglucanase (EC. 3.2.1.4) produced, can be employed for this intention. The degradation of cellulosic substances is due to a complex enzymatic system called cellulase. Most of these substances displayed nonspecific hydrolysis towards chitosan substrates. In addition, endoglucanase production has been proclaimed to have chitosan hydrolysis activity [156]. *Streptomyces* sp. and *Bacillus* sp. cellulase also showed chitosanase activity [157].

Innumerable chitosanases have been isolated from microorganisms [155], including fungi, bacteria and Actinomycetes. *Bacillus* sp. FERM-P-8139 is a mutant isolated from soil that produces a potent extracellular chitosanase when seeded into the chitosan colloid as an inducible substance. The purified chitosanase can be used for the preparation of glucosamine oligomers having antibacterial and antifungal activities [158].

## 5. Synergistic Effect of Chitin/Chitosan and Endophytic Bacteria (Genus *Bacillus* sp.) on the Germination of Seeds, Growth and Fructification of Plants

In view of the fact that the size of polymers limits their use as promoters of plant growth and development of endophytic bacteria with chitinolytic activity guarantees the degradation of polymers, therefore facilitating the absorption of degradation products by plants [83].

Ergo, the inquiry by Ortiz-Rodriguez et al. (2010) [159] showcased that the use of *Bacillus thuringiensis* strain produced an endochitinase that allows growth of the bacteria on a medium rich in chitin, which generates the oligosaccharides derived from chitin that have an impact on the growth of plants.

Das et al. (2010) [160] noticed that the bacterization of peanut seeds with *Paenibacillus elgii*, in the presence of chitosan, noticeably enhanced the germination percentage (75%), the length of the shoot (25.33%), root (31.75%), fresh weight (31.75%), dry weight 61.32%, and total chlorophyll content (7.87%).

Besides, production of chitin-binding protein (Cry) by a specific strain of *Bacillus thuringiensis* reinforced the insecticidal and fungistatic activity of these proteins [161].

Therefore, Nadège et al. (2016) [162] have established the beneficial effect of the combination of chitinolytic bacteria and chitosan on the growth of corn seeds treated with chitosan and a bacterial solution of *Azospirillumli poferum* and *Pseudomonas fluorescens*. The corn seeds featured an increase in vigor index with 36.44% compared to the control. Whereas, *Pseudomonas putida* significantly improved the weight of roots with 44.84% and the weight of germinated seeds (31.39%), though chitosan and *Pseudomonas putida* augmented the weight of the shoots (65.67%) [162].

Besides inducing chitinase activity and promoting bacterial growth, the addition of chitin has also been shown to have other beneficial effects on bacteria [163]. The combination between chitosan and endophytic bacteria amplified the vigor index of corn seeds (*Zea mays* L.) by more than 36.44% compared to the control [162]. It is worth mentioning that in the powder state, chitin and chitosan persist in assimilation by seedlings due to their large size. While as a monomer, they become effortlessly exploitable as a nutrient rich in carbon, oxygen and nitrogen. This can only be done through chemical or biological degradation.

The study by Kumar et al. (2019) [164] exposes that the sorghum seeds bacterized with four bacterial strains (possessing chitinolytic characteristics), and treated or not with chitosan (different deacetylation degree), leads to higher seedling growth, which is expressed by high radical lengths of 25,9 cm, the highest hypocotyl length of 32,1 cm, and the mean dry weight of 132,7 mg per plant.

On account of this, the study by Nadège et al. (2016) [162] corroborates our hypothesis on the beneficial effect of the combination of chitinolytic bacteria and chitosan on plant growth. For, maximum heights of 17.66% were obtained by corn plants treated with a solution of chitosan and strains of *Azospirillum lipoferum*, *Pseudomonas fluorescens*, *Pseudomonas putida,* and *Pseudomonas fluorescens*. Over and above that, this combination induced the greatest boost in leaf production per plant (50.09%) the weight of the aerial (84.66%) and underground (108.77%) part. Plants inoculated with *Azospirillum lipoferumont* exhibited a large leaf area with an increase of 54.08%, while, the synergic effect of *P. putida, P. fluorescens* and chitosan increased the dry weight by 26.35% and 18.18% of the aerial and root parts of corn plants. Moreover, the simultaneous presence of chitosan and *P. fluorescens, P. putida,* A. *lipoferum* increase the nitrogen content of plants by 41.61%.

Moreover, the trials of Narandelger et al. (2015) [165] on plants of *Lycopersicon esculentum* L. indicated that the synergy amidst biofertilizers (endophytic bacteria) and chitosan had an impact on the control of diseases due to the presence of *Fusarium* spp., not to mention the effect on the yield of the greenhouse tomato. These researchers proved that this combination had an influence on the number and weight of fruits per plant. For the reason that after two weeks the plants had reached an average number of 26 fruits, with an typical weight of 1252 g compared to the control without biofertilizers, which registered a number of 10 fruits and a weight of 575 g.

These advantageous effects on bacterial endophytes are caused by the alterations of the physiology of the host plant [94]. The inoculation of endophytic bacteria, namely *Acinetobacter johnsonii*, *Chryseobacterium indologene* and *Bacillus pumilus* into *Beta vulgaris* increase the chlorophyll content of plants, which generates advanced production of carbohydrates versus the uninoculated plants [121].

## 6. Effects of Formulation Chitin/Chitosan and *Bacillus* sp. on Plant Protection

Today, the control of pesticides is a central public action issue in the field of environmental health. Even in the presence of the rules governing their marketing and used in agriculture, fluctuations continue to appear on the horizon. Biological control is one of the promising methods; it consists of employment of antagonistic microorganisms and/or naturel products (biopolymers, essentials oils…) [166].

The study conducted by Kishore and Pande (2007) [167] on the reduction of the severity of *Botrytis cinerea* in chickpea showed the beneficial effect of using the foliar spray of *Bacillus circulans* CRS 7 with colloidal chitin on the improvement of the inhibitory effect of this isolate on the onset of symptoms, utilizing a scale ranging from 1 to 9. The authors found that disease severity was reduced from 9 in control plants to 4.4 and 4.1 with the presence of *Bacillus circulans* CRS 7 and colloidal chitin at 0.5% and 1% (*w/v*), respectively.

In addition, the combination of sol amendment by 0.5% chitin and *B. licheniformis* LS674 or *B. subtilis* HS93 significantly reduced pepper root rot caused by *Phytophthora* by 70% and 64%, respectively. Thus, it had a remarkable action on root rot caused by *Rhizoctonia*, which resulted in a difference of 50% and 62% compared to the control of this plant [168].

According to the experiment conducted by Manjula and Podile (2005) [169], 4-month cultivation of pigeon pea plants (inoculated with *Aspergillus niger*) in peat supplemented with 0.5% of chitin and *Bacillus subtilis* AF1 increased emergence and dry weight of pigeon pea seedlings by 29% and 33%, compared to an increase of 21% and 30%, respectively, for *Bacillus subtilis* AF1 alone.

The application of chitinolytic bacteria in the control of pathogens depends on their chitinase activities. The main factor, influencing the synthesis of chitinolytic enzymes is the availability of chitin substrates, which will initiate the expression of the chitinase gene. Several studies have shown that the best substrates for chitinase synthesis are colloidal chitin and shrimp shell powder, however, colloidal chitin is the most widely used. Shrimp waste has also been used as a substrate for the synthesis of chitinases [170].

## 7. Conclusions

Numerous studies have demonstrated the advantageous effects of chitin and/or chitosan on the stimulation of protection and crop growth/development. These latter are remarkably linked to the physico-chemical properties of these biopolymers extracted from the shrimp shells. Nevertheless, the combination of the biopolymers and the bacterial strains of the genus *Bacillus* sp. that have tremendous biochemical and enzymatic characteristics, has been exposed to be beneficial for the control of phytopathogens and the improvement of plant growth and fructification. The action of treatments based on chitin, chitosan and endophytic bacteria is revealed to be analogous to that acquired with pesticides and synthetic fertilizers. This efficiency will be employed to outline a path for the development of products based on biopolymers and endophytic bacteria.

## Figures and Tables

**Table 1:** Chitin and chitosan sources [13].

	Source	Chitin Percentage
**Chitin**	Crab	10
	Majoidea (Spider crab)	16
	Lobster	17
	Cuttlefish	20
	Shrimp	22
	Sea mantis	24
	Lobster	32
	Crayfish	36
	Squid	40
**Chitosan**	Marbled crab (*Grapsus marmoratus*)	10
	Edible crab	70
	Grasshopping Lobster (*Scyllarus arctus*)	25
	Crawfish (*Palinurus vulgaris*)	32
	Prawns (*Palaemon fabricius*)	44
	Squid (*Loligo vulgaris*)	40
	Mushrooms	
	*Mucor rouxii*	9.4
	*Aspergillus niger*	42
	*Aspergillus phoenicis*	23.7

## Data Availability

Data sharing not applicable.
